# Attenuated accumulation of jasmonates modifies stomatal responses to water deficit

**DOI:** 10.1093/jxb/ery045

**Published:** 2018-02-08

**Authors:** Carlos De Ollas, Vicent Arbona, Aurelio Gómez-Cadenas, Ian C Dodd

**Affiliations:** 1 Departamento de Ciencias Agrarias del Medio Natural. Universitat Jaume I, Spain; 2 Lancaster Environment Centre, Lancaster University, Lancaster, UK

**Keywords:** Drought stress, JA, ABA, grafting, *Solanum lycopersicum*

## Abstract

To determine whether drought-induced root jasmonate [jasmonic acid (JA) and jasmonic acid-isoleucine (JA-Ile)] accumulation affected shoot responses to drying soil, near-isogenic wild-type (WT) tomato (*Solanum lycopersicum* cv. Castlemart) and the *def-1* mutant (which fails to accumulate jasmonates during water deficit) were self- and reciprocally grafted. Rootstock hydraulic conductance was entirely rootstock dependent and significantly lower in *def-1*, yet *def-1* scions maintained a higher leaf water potential as the soil dried due to their lower stomatal conductance (*g*_s_). Stomatal sensitivity to drying soil (the slope of *g*_s_*versus* soil water content) was low in *def-1* self-grafts but was normalized by grafting onto WT rootstocks. Although soil drying increased 12-oxo-phytodienoic acid (OPDA; a JA precursor and putative antitranspirant) concentrations in *def-1* scions, foliar JA accumulation was negligible and foliar ABA accumulation reduced compared with WT scions. A WT rootstock increased drought-induced ABA and JA accumulation in *def-1* scions, but decreased OPDA accumulation. Xylem-borne jasmonates were biologically active, since supplying exogenous JA via the transpiration stream to detached leaves decreased transpiration of WT seedlings but had the opposite effect in *def-1*. Thus foliar accumulation of both ABA and JA at WT levels is required for both maximum (well-watered) *g*_s_ and stomatal sensitivity to drying soil.

## Teaser

Drought-induced stomatal closure correlates with JA accumulation, but was unaffected in reciprocal grafts of wild-type and *def-1* mutant (does not accumulate JA) plants. Only stomata of *def-1* self-grafts were relatively drought insensitive.

## Introduction

Jasmonic acid (JA) and other members of the oxylipin pathway such as 12-oxo-phytodienoic acid (OPDA) and the bioactive jasmonic acid-isoleucine (JA-Ile; Katsir *et al.*, 2008; Fonseca *et al.*, 2009) have been associated with tolerance to biotic stresses (Wasternack and Hause, 2013) by mediating defence against herbivores and necrotrophic pathogens. JA and its derived molecules (mainly JA-Ile as the bioactive interactor with the COI1 receptor) are known as jasmonates, a subgroup of oxylipins derived from OPDA also associated with the systemic response to physical stresses such as wounding (Sun *et al.*, 2011). Nevertheless, they can also be involved in tolerance to other abiotic stresses such as drought and salinity (Cheng *et al.*, 2013; Alam *et al.*, 2014; Savchenko *et al.*, 2014; Kazan, 2015). Water deficit increases tissue JA concentrations in several species (Creelman and Mullet, 1995; Mahouachi *et al.*, 2007; Brossa *et al.*, 2011; Chen *et al.*, 2016), as well as JA-Ile (De Ollas *et al.*, 2015), while mutations in several steps of the JA biosynthesis and signalling pathways alter tolerance to water deficit (Harb *et al.*, 2010; Grebner *et al.*, 2013; Riemann *et al.*, 2015). Although JA and abscisic acid (ABA) seem to influence each other’s biosynthesis (Bandurska *et al.*, 2003; Kim *et al.*, 2009; De Ollas *et al.*, 2013; Fragoso *et al.*, 2014), few studies have assessed their relative importance in mediating physiological responses to water deficit.

Although ABA plays a central role in regulating stomatal closure in response to soil drying (Raghavendra *et al.*, 2010; Lee and Luan, 2012), other phytohormones can also influence this response. Exogenous application of either ABA or methyl jasmonate (MeJA) elicited similar stomatal closure using the same second messengers (Munemasa *et al.*, 2011). The oxylipin OPDA is claimed to promote stomatal closure more effectively and independently from JA and in co-operation with ABA (Savchenko *et al.*, 2014). JA/JA-Ile-dependent stomatal regulation may also be important since applying ABA or MeJA to the Arabidopsis *jar1-1* mutant (which is defective in conjugating isoleucine to JA) elicited less stomatal closure than in wild-type (WT) plants (Suhita *et al.*, 2004). Interestingly, the Arabidopsis *aos* (JA/JA-Ile and OPDA deficient) and the *opr3* (deficient in JA/JA-Ile but not OPDA) mutants had higher and lower stomatal aperture than WT plants, respectively (Savchenko *et al.*, 2014). Thus various oxylipins and ABA may be involved in regulating stomatal responses of plants in drying soil.

While these studies explored stomatal responses of isolated epidermal peels (reviewed in De Ollas and Dodd, 2016), few studies have examined JA/JA-Ile effects on whole-plant transpiration. While leaf scorching increased JA concentration concurrent with stomatal closure in distal leaves (Hlaváčková *et al.*, 2006), withholding water from the WT and the Arabidopsis *aos* mutant (JA/JA-Ile and OPDA deficient) elicited similar stomatal closure (Brossa *et al.*, 2011). Similar drought-induced stomatal closure in genotypes varying in oxylipin status is consistent with a model where stomatal behaviour is primarily determined by plant hydraulics (Tardieu, 2016). In this context, exogenous application of ABA (reviewed in Dodd, 2013) or MeJA (Sánchez-Romera *et al.*, 2014) increased root hydraulic conductivity (L_pr_) along with gene expression of several aquaporins (Sánchez-Romera *et al.*, 2014). Moreover, a tomato (*Solanum lycopersicum*) mutant (*def-1*) defective in stress-induced JA biosynthesis had lower L_pr_, which was restored by MeJA treatment (Sánchez-Romera *et al.*, 2014). Although phytohormone-mediated variation in L_pr_ may influence leaf water relations, whether this regulates stomatal responses to soil drying is less clear. Reciprocal grafting with different root and scion genotypes determined the role of long-distance JA/JA-Ile signalling in wound responses (Fragoso *et al.*, 2014), but the impact of variation in root and shoot jasmonates on plant water relations responses to soil drying has not been assessed.

Since both ABA and JA can regulate both L_pr_ and stomatal conductance (*g*_s_), JA may alter physiological responses to drying soil. Initially, ungrafted plants of two tomato mutants compromised in the oxylipin pathway [*spr2*, constitutively deficient in the biosynthesis of OPDA, JA, and the bioactive JA-Ile (Li *et al.*, 2003); and *def-1*, with diminished stress-induced JA/JA-Ile accumulation (Howe *et al.*, 1996)] were grown under water deficit to characterize root and shoot phytohormonal responses. Then, to better understand the involvement of both scion- and root-sourced jasmonates in physiological responses to water deficit, self- and reciprocally grafted WT and *def-1* mutant (with attenuated JA accumulation in drying soil) plants were grown in well-watered and drying soil. Physiological responses such as whole-plant transpiration, leaf and root water potential, L_pr_, and *g*_s_ were correlated with leaf and xylem sap ABA and oxylipin (OPDA and JA/JA-Ile) concentrations. To determine whether xylem-supplied phytohormones regulate stomatal responses, detached leaves were supplied with JA or ABA via the transpiration stream (Zhang and Davies, 1991). We tested the hypothesis that jasmonates can regulate transpiration via local (foliar jasmonates accumulation) but also long-distance (root to shoot transport through the transpiration stream) processes.

## Materials and methods

### Plant material

To characterize jasmonate effects on plant responses to water deficit, two tomato (*S. lycopersicum*) mutants (*def-1* and *spr2*) were grown under well-watered and water-limited conditions. The (unidentified) *def-1* mutation (originally described as JL5; Howe *et al.*, 1996) prevents JA (and hence JA-Ile) accumulation under wounding and biotic stress, but basal JA/JA-Ile concentrations are similar to those of its near-isogenic line Castlemart. This genetic lesion is not related to any identified biosynthetic gene and this mutant is not compromised in OPDA biosynthesis (Howe *et al.*, 2000). Thus *def-1* has attenuated stress-induced JA/JA-Ile accumulation.

In the *spr2* (suppressor of prosystemin-mediated responses 2; Howe *et al.*, 1996) mutant, the lack of a chloroplast fatty acid desaturase in the octadecanoid pathway compromises constitutive JA biosynthesis, severely decreasing leaf (Li *et al.*, 2003) and root (Tejeda-Sartorius *et al.*, 2008) JA levels. Wound-induced JA biosynthesis is also impaired (Li *et al.*, 2003) as is OPDA biosynthesis (Wasternack *et al.*, 2012). Thus *spr2* is more generally deficient in both jasmonates and OPDA biosynthesis.

### Experiments with ungrafted plants

Seeds of the WT, *spr2*, and *def-1* were sown individually in 1 cm diameter plastic pots filled with a mixture of peat/perlite (80/20, v/v) and covered with black plastic to ensure high humidity and darkness to promote germination. After 4–6 d, the plastic was removed to prevent seedling etiolation. After a further week, seedlings were potted into individual 216 cm^3^ pots and watered daily for a further 2 weeks, then transplanted to 4000 cm^3^ pots filled with the same substrate. After another week under well-watered conditions, pots were covered with plastic bags to limit soil evaporation. Randomly selected plants of each genotype were divided into well-watered (daily replacement of evapotranspiration) and water deficit [soil water content (SWC) allowed to drop to <0.2–0.15 cm^3^ cm^–3^ before re-watering] groups. After 5 d of soil drying when all genotypes were at a similar SWC, whole-plant transpiration and water potential of the youngest fully expanded leaf (Ψ_leaf_) were measured as described below, with xylem sap collected from this leaf. Prior to measuring Ψ_leaf_, the next youngest leaf was excised and placed in liquid N_2_. Then the roots were carefully washed from the substrate, and placed in liquid N_2_.

### Experiments with grafted plants

Seeds of WT and *def*-1 tomato were sown individually in 1 cm diameter plastic pots filled with Levington M3 compost (Scotts Company Ltd, Ipswich, UK) and covered with black plastic to ensure high humidity and darkness to promote germination. After 4–6 d, the plastic was removed to prevent seedling etiolation. After a further week, seedlings were potted into individual 500 cm^3^ pots and watered daily for a further 2 weeks prior to grafting. Graft unions were established just below the cotyledonary node as previously described (Dodd *et al.*, 2009). Two weeks after grafting, plants were transplanted to pots containing an organic loam (John Innes No. 2, UK), for which a moisture release curve has been previously published (Dodd *et al.*, 2010). Pots were cylinders, 6.5cm in diameter and 23cm in height (750cm^3^ volume), with stainless steel mesh (0.7mm aperture) at the base to assist drainage, and designed to fit tightly in a pressure chamber of the same volume (Soil Moisture Equipment Corp., Santa Barbara, CA, USA). The tops of the pots were taped (Advance Tapes, Leicester, UK) to reduce evaporation from the soil surface. Plants were watered immediately following transplanting and daily thereafter, and allowed to establish for 1 week. Plants were harvested 23–27d post-grafting. All plants were watered to drained capacity every day until 2d before measurements. Pots were placed on a saucer in a walk-in controlled environment room with a day/night temperature of 22/16 °C and a 12 h photoperiod (06.00–18.00 h). Day/night relative humidity was 42/54%, CO_2_ concentration was 440/390 ppm, and light intensity at plant height was between 400 µmol m^−2^ s^−1^ and 640 µmol m^−2^ s^−1^ photosynthetic photon flux density (PPFD). The day before harvesting, some randomly chosen plants were watered to drained capacity while others were not watered to impose a soil drying treatment. Preliminary trials showed that transpiration reduced SWC from drained capacity (0.5 cm^3^ cm^–3^) to low soil moisture (0.2 cm^3^ cm^–3^) within 24–48 h depending on the graft combination. Plants were harvested (09.00–14.00 h) in three blocks with one plant of each graft combination per block.

### Physiological measurements

Transpiration was calculated by weighing the pots daily (09.00–10.00 h), with evaporation from the soil surface ignored. Transpiration was normalized to whole-plant leaf area, which was measured with a Li-3100 Area Meter (Li-Cor Inc., Lincoln, NE, USA).

Immediately prior to harvest, stomatal conductance (*g*_s_) was measured on the middle leaflet of a fully expanded leaf (fourth or fifth leaf from the top of the plant) using infra-red gas analysis (6400xt Li-Cor Portable Photosynthesis System, Lincoln, NE, USA). Cuvette conditions approximately matched the environmental conditions within the controlled-environment room: CO_2_ at ambient levels (390 ppm), 600 μmol m^−2^ s^−1^ PPFD, cuvette temperature of 22 °C, and ambient humidity typically 40–50%.

Immediately after measuring *g*_s_, leaf water potential (Ψ_leaf_) was measured on the same leaf using a pressure chamber (Model 3000F01 Plant Water Status Console; Soil Moisture Equipment Corp.). Detached leaves were transported to the laboratory and placed in the pressure chamber within 60 s of excision. Once in the chamber, the cut petiole surface was cleaned with deionized water and filter paper to remove cellular debris. Pressure was raised in the chamber at a rate of 0.02 MPa s^−1^, and Ψ_leaf_ was recorded when xylem sap collected on the cut surface. An overpressure of 0.4 MPa was then applied to each leaf to collect xylem sap. The initial droplets of sap were discarded, then sap was sampled for 2–3 min using a pipette. Xylem sap samples were stored in a previously weighed 1.5 ml microfuge tube.

Root water potential (Ψ_root_) was measured after Ψ_leaf_ by de-topping the plants with a razor blade 2 cm below the graft union and introducing the whole pot into the pressure chamber. The cut stem was cleaned with deionized water and filter paper to remove cellular debris. Pressure was raised in the chamber at a rate of 0.01 MPa s^−1^, and Ψ_root_ was recorded when xylem sap collected on the cut surface. An overpressure of 0.2 MPa was then applied to collect xylem sap from the roots. In case natural exudation occurred following shoot removal, Ψ_root_ was considered equal to zero. After measuring Ψ_root_, the soil was weighed and then placed in a tray in a drying oven. SWC was calculated with the formula [SWC=(m_wet_–m_dry_)/m_dry_] where m_wet_ is the soil fresh weight when harvested, and m_dry_ is the weight of the dry soil after 3 d in an oven at 80 °C.

### Root hydraulic conductivity measurements

Three plants of each graft combination were randomly selected to measure root hydraulic conductivity (L_pr_). After shoot removal, a series of overpressures (from 0.1 MPa to 0.6 MPa in 0.1 MPa increments) were applied and the sap flow rate determined at each pressure. Roots were washed from the pots and kept moistened with wet paper, before scanning (Epson perfection v700 photo), with the scanner connected to a computer running the WinRHIZO™ software (Regent Instruments, Canada). Root hydraulic conductivity quantifies root permeability to the flow of water, by applying increasing pneumatic pressure to the root zone. Applied pressure and water flow are linearly correlated, with the slope of the relationship defined as the root hydraulic conductance (when data are normalized to root surface area).

### Detached leaf transpiration assays

Seeds of the *def-1* and *spr2* mutants and their corresponding WT (cv. Castlemart) were sown as described above, and then potted into individual 350 cm^3^ pots filled with a mixture of peat/perlite (80/20, v/v). These were placed in a controlled-environmental chamber with a day/night temperature of 25/16 °C and a 16 h photoperiod (07.00–23.00 h), and watered every 2–3 d. Day/night relative humidity was 42/54%, CO_2_ concentration was 450/380 ppm, and light intensity at plant height between 400 µmol m^−2^ s^−1^ and 500 µmol m^−2^ s^−1^ PPFD. Plants were kept in those conditions for a further 3 weeks prior to the experiments.

Well-watered plants (6 weeks old, irrigated to drained capacity) were kept in the dark overnight. A razor blade severed fully expanded leaves at the petiole–stem junction, which were then recut (5 mm from the initial excision site) under deionized water to prevent xylem embolism. The leaves were immediately transferred to a 20 ml glass vial, containing 15 ml of artificial xylem sap (the same composition as in Dodd *et al.*, 2003), and placed in a dark room for 2 h. The top of the glass vial was sealed by parafilm, with a small hole to insert the petiole in the artificial xylem sap (whilst reducing evaporative losses). After 2 h, all leaves were randomly transferred to glass vials containing 15 ml of artificial xylem sap with different ABA or JA concentrations (0, 10, 100, and 1000 nM). After these preliminary dose–response curves, further experiments used 1000 nM as the minimum concentration of JA that significantly decreased transpiration of WT plants.

Leaves were then randomized within the controlled-environment room with conditions described in the previous paragraph. Each vial was weighed initially by a four-point analytical balance, then re-weighed hourly for 5 h. Leaves that showed any (wilting) symptoms of embolism (<10% of the total) were discarded. Finally, leaf area was recorded using a scanner and the Easy Leaf Area software to normalize the transpiration rate for leaf area. After the assay, petioles were gently rinsed with distilled water to remove any of the feeding solution, the leaf was placed in a pressure chamber, and xylem sap (100 µl per sample) was collected at a fixed overpressure (0.1–0.2 MPa) above balancing pressure. Leaves were then frozen in liquid N_2_ to analyse the hormone concentrations.

To determine possible wounding-related hormonal changes caused by excising leaves for use in the transpiration assay, comparable leaves were collected from intact plants grown under the same conditions, both before (pre-excision control) and after the transpiration assay (post-excision control). These leaves were immediately placed in the pressure chamber to collect xylem sap as described above, then frozen in liquid N_2_ to analyse hormone concentration.

### Phytohormone quantification

ABA, JA, OPDA, and JA-Ile were extracted and analysed essentially as previously described (De Ollas *et al.*, 2013) with slight modifications. Briefly, 0.2 g of dry plant material was extracted in 2 ml of distilled H_2_O after spiking with 25 μl of a 2 mg l^−1^ solution of d^6^-ABA and dihydrojasmonic acid (DHJA) as internal standards. After centrifugation (10 000 *g* at 4 °C), supernatants were recovered and the pH was adjusted to 3.0 with 30% acetic acid. The acidified water extract was partitioned twice against 3 ml of di-ethyl ether. The organic layer was recovered and evaporated under vacuum in a centrifuge concentrator (Speed Vac, Jouan, Saint Herblain Cedex, France). The dry residue was then re-suspended in a 9:1 H_2_O:MeOH solution by sonication. The resulting solution was filtered and directly injected into a UPLC system (Waters Acquity SDS, Waters Corp., Milford, MA, USA) interfaced to a TQD triple quadrupole (Micromass Ltd, Manchester, UK) mass spectrometer through an orthogonal Z-spray electrospray ion source. Xylem sap (75 µl) was spiked with 10 µl of internal standard solution and diluted with deionized water to a total volume of 200 µl; the solution was filtered and injected into the UPLC system using the same conditions as used for tissue extraction.

Separations were carried out on a Gravity C18 column (50 × 2.1 mm, 1.8 μm, Macherey-Nagel GmbH, Germany) using a linear gradient of MeOH and H_2_O supplemented with 0.1% acetic acid at a flow rate of 300 μl min^−1^. Transitions for ABA/d^6^-ABA (263>153/269>159), JA/DHJA (209>59/211>59), OPDA (291>165), and JA-Ile (322>130) were monitored in negative ionization mode. Quantitation of plant hormones was achieved by external calibration with known amounts of pure standards using Masslynx v4.1 software.

### Stomatal density and size analysis

Epidermal imprints were taken from fully expanded leaves following a similar protocol to that of Scalschi *et al.* (2015). Leaflets were placed on glass slides with the abaxial epidermis in contact with dental resin. For stomatal analysis, images of randomly selected regions were taken using a Leica IRB microscope equipped with a Leica DC300F camera (Leica Microsystems CMS GmbH, Wetzlar, Germany). Stomatal number and size of at least 30 random sections per genotype were calculated with the ImageJ software. Size or stomatal area was calculated assuming the area of an ellipse.

### Statistical analysis

**Fig. 1.**
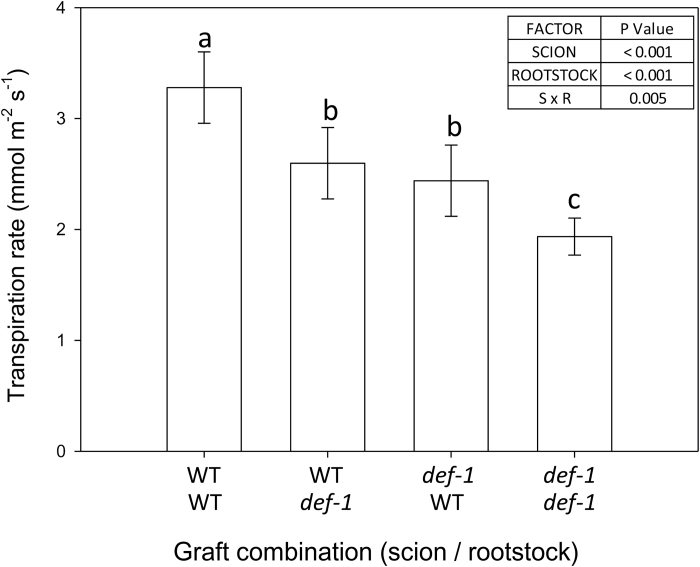
Transpiration rate of wild-type (WT) and *def-1* self- and reciprocal grafts (top scion, bottom rootstock), normalized to whole-plant leaf area. Bars represent the mean ±SE of eight replicates per graft combination; different letters denote significant (*P*<0.05) differences between graft combinations. The table summarizes the significance (*P*-values) of scion, rootstock, and their interaction (S×R) after ANOVA.

**Fig. 2.**
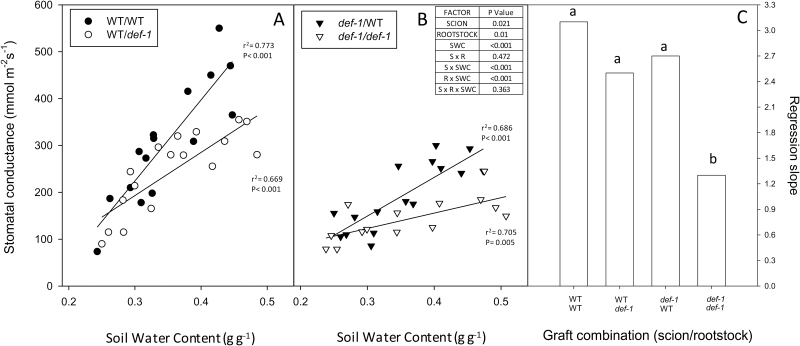
Stomatal conductance (*g*_s_) versus soil water content (SWC) of (A) Self-grafted wild-type (WT) plants (filled circles) and WT scions grafted onto *def-1* rootstocks (open circles) and (B) self-grafted *def-1* plants (open triangles) and *def-1* scions grafted onto WT rootstocks (filled triangles). (C) Slope of the regression line between relative *g*_s_ (normalized to maximum *g*_s_) and SWC of the four graft combinations, with different letters showing significant differences in slope. In (A) and (B), each point is an individual plant with linear regression lines fitted for each graft combination with *r*^2^ and *P*-values reported. The table summarizes the significance (*P*-values) of scion, rootstock, SWC, and their interactions after ANOVA.

**Fig. 3.**
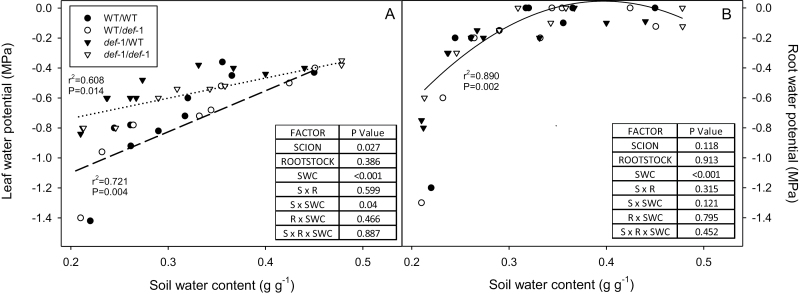
(A) Leaf and (B) root water potential versus soil water content (SWC) of self-grafted wild-type (WT) plants (filled circles), *def-1* self-grafts (open triangles) and reciprocal graft (scion/rootstock) combinations (WT/*def-1*, open circles; and *def-1*/WT, filled triangles). Each point is an individual plant, with lines defining significant correlations of (A) WT (dashed line) and *def-1* (dotted line) scions (linear regressions) and (B) all plants (second-order regression) with *r*^2^ and *P*-values reported. The tables summarize the significance (*P*-values) of scion, rootstock, SWC, and their interactions after ANOVA.

**Fig. 4.**
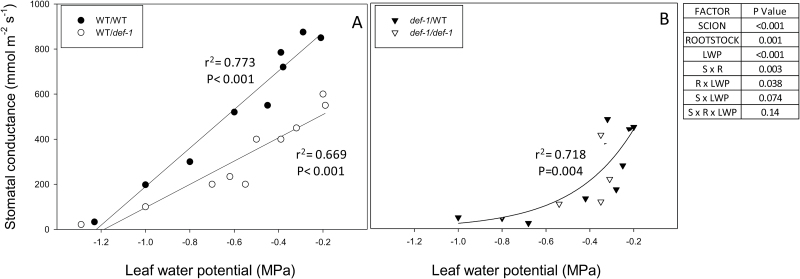
Stomatal conductance versus leaf water potential (LWP) of (A) self-grafted wild-type (WT) plants (filled circles) and WT scions grafted onto *def-1* rootstocks (open circles) and (B) self-grafted *def-1* plants (open triangles) and *def-1* scions grafted onto WT rootstocks (filled triangles). Each point is an individual plant, with lines defining significant correlations of (A) WT self-grafts and WT/*def-1* plants (linear regression) and (B) *def-1* scions [exponential growth model (*y*=*ax*^b^) with *r*^2^ and *P*-values reported. The table summarizes the significance (*P*-values) of scion, rootstock, LWP, and their interactions after ANOVA.

**Fig. 5.**
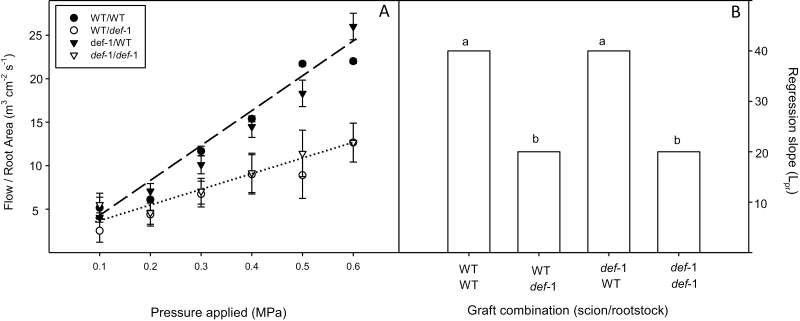
(A) Root xylem sap flow rate (normalized to root area) versus applied pressure for detached roots of self-grafted wild-type (WT) plants (filled circles), *def-1* self-grafts (open triangles), and reciprocal graft (scion/rootstock) combinations (WT/*def-1*, open circles; and *def-1*/WT, filled triangles). Symbols are the mean ±SE of three replicates. Lines denote a significant correlation between WT (dashed line) and *def-1* (dotted line) rootstocks. (B) Root hydraulic conductance (the slope of flow rate versus applied pressure) of the different graft combinations. Letters denote a significant (*P*<0.05) difference between graft combinations after Duncan’s HSD.

**Fig. 6.**
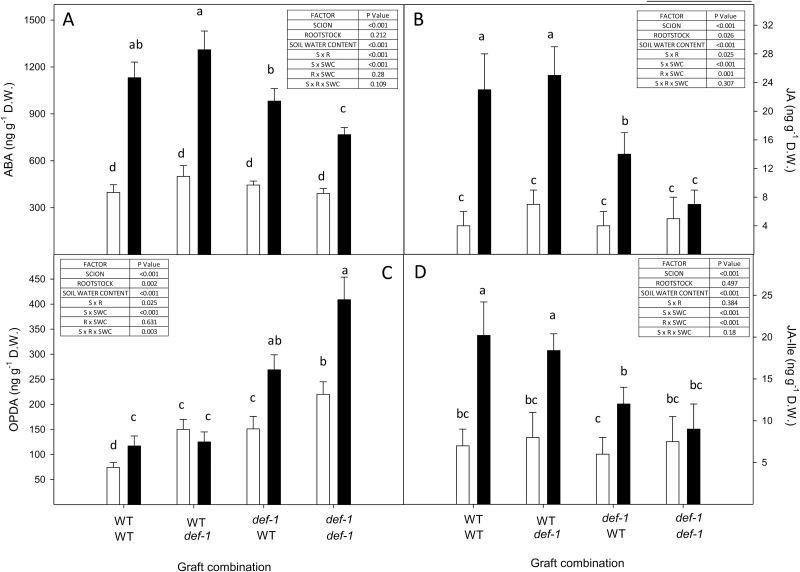
Foliar ABA (A), JA (B), OPDA (C), and JA-Ile (D) concentrations of wild-type (WT) and *def-1* self-grafts and reciprocal graft (scion/rootstock) combinations under high (0.3<SWC<0.5 cm^3^ cm^–3^) and low (0.2<SWC<0.3 cm^3^ cm^–3^) soil water content (SWC), represented by open and filled bars, respectively. Bars are the mean ±SE of four replicates, with different letters denoting significant (*P*<0.05) differences between graft combinations after Duncan’s HSD within a panel. The tables summarize the significance (*P*-values) of scion, rootstock, SWC, and their interactions after ANOVA.

**Fig. 7.**
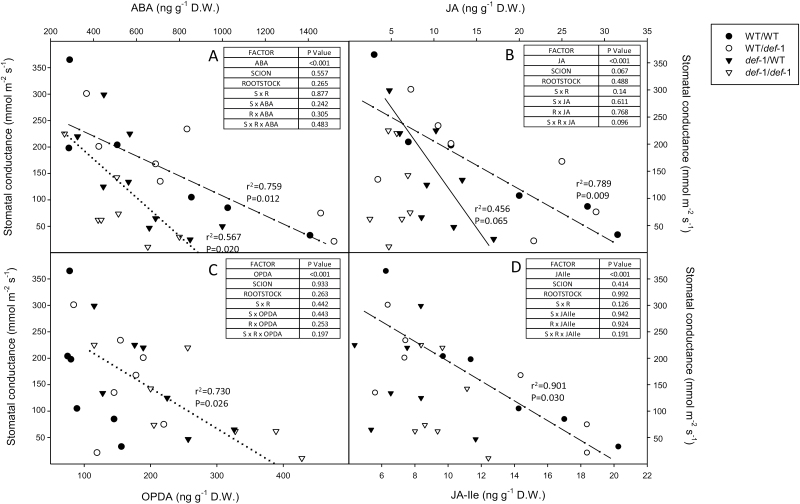
Stomatal conductance versus foliar ABA (A), JA (B), OPDA (C), and JA-Ile (D) concentrations in wild-type (WT) self-grafts (filled circles), *def-1* self-grafts (open triangles), and reciprocal graft (scion/rootstock) combinations (WT/*def-1*, open circles; and *def-1*/WT, filled triangles). Correlations between hormone concentration and stomatal conductance reported (*r*^2^ and *P*-values) for WT scions (dashed lines), *def-1* scions (dotted lines), and *def-1*/WT plants (plain line). The tables summarize the significance (*P*-values) of hormones, scion, rootstock, and their interactions after ANOVA.

**Fig. 8.**
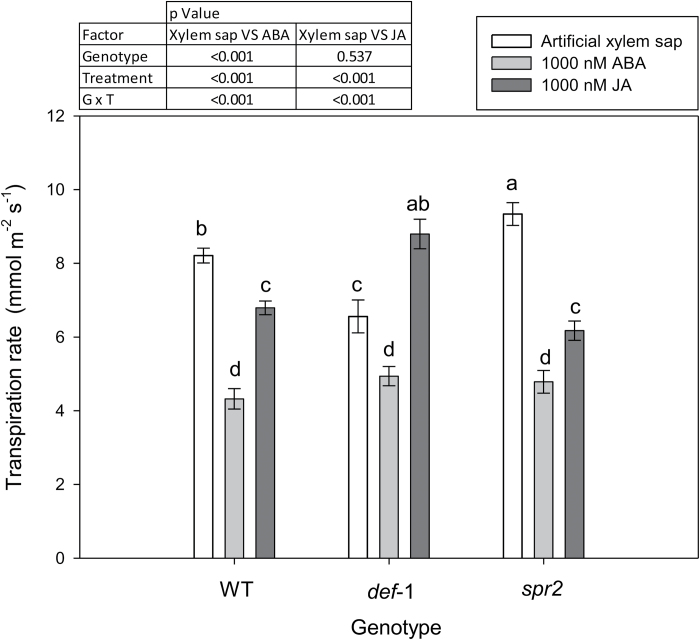
Transpiration rate of detached wild-type (WT), *def-1*, and *spr2* leaves fed with artificial xylem sap (open bars), 1000 nM ABA (light grey bars), or 1000 nM JA (dark grey bars). Symbols are the mean ±SE of four replicates, with different letters denoting significant differences between all genotype/treatment combinations. The table summarizes the significance (*P*-values) of genotype, treatment, and their interaction after ANOVA of pairwise treatments (artificial xylem sap versus 1000 nM ABA and artificial xylem sap versus 1000 nM JA).

**Fig. 9.**
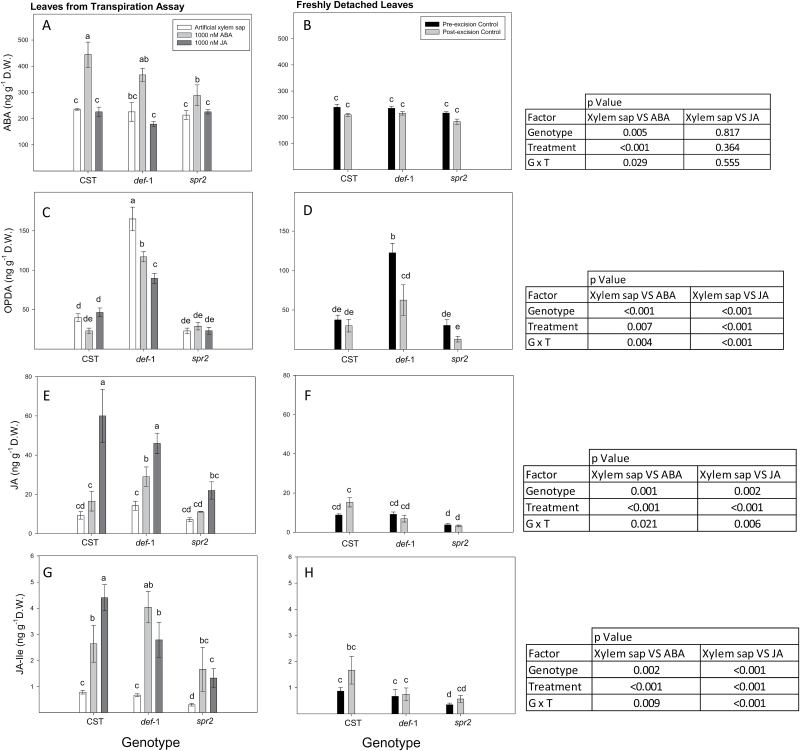
ABA (A, B) OPDA (C, D), JA (E, F), and JA-Ile (G, H) concentrations of WT, *def-1*, and *spr2* leaves after 5 h of xylem feeding with artificial xylem sap (open bars), 1000 nM ABA (light grey bars), or 1000 nM JA (dark grey bars) (left panels) or freshly detached leaves before (filled bars) and 5 h after (grey bars) starting the transpiration assay (right panels). Bars are the mean ±SE of four replicates, with different letters denoting significant differences between treatments after Duncan’s HSD, comparing across both panels. The tables summarize the significance (*P*-values) of genotype, treatment, and their interactions after ANOVA of pairwise treatments (artificial xylem sap versus 1000 nM JA and artificial xylem sap versus 1000 nM ABA).

In ungrafted plants (Table 1) and the detached leaf experiments (Figs 8, 9), genotype and treatment effects (and their interaction) were determined using two-way ANOVA, with Duncan’s HSD (*P*<0.05) used to determine significant differences between genotype/treatment combinations. In grafted plants, rootstock and scion effects (and their interaction) were determined using two-way ANOVA (e.g. Fig. 1), with significant effects of graft combinations on the relationships between soil and plant variables determined by analysis of covariance (ANCOVA; e.g. Figs 2–7). Differences in detached leaf transpiration and foliar phytohormone concentrations (Figs 8, 9) between genotype/treatment combinations were determined using one-way ANOVA, with significant (*P*<0.05) differences determined using Duncan’s HSD.

**Table 1.** Whole-plant transpiration, leaf area, leaf water potential, and ABA, OPDA, JA, and JA-Ile concentrations in leaves, xylem sap, and roots of the wild type (WT), *def-1*, and *spr2* under well-watered (WW) and water deficit (WD) conditions

	Wild type (WT)		*def-1*		*spr2*		*P*-values from two-way ANOVA		
	WW	WD	WW	WD	WW	WD	Genotype	Treatment	G×T
Whole-plant transpiration (mmol m^–2^ s^–1^)	2.18 ± 0.06 a	0.70 ± 0.06 c	1.41 ± 0.05 b	0.45 ± 0.12 c	2.50 ± 0.15 a	0.77 ± 0.45 c	<0.001	<0.001	0.006
Leaf area (cm^2^)	430 ± 32 a	416 ± 25 a	378 ± 24 a	385 ± 15 a	330 ± 36 b	325 ± 19 b	0.003	0.771	0.802
Leaf water potential (MPa)	–0.30 ± 0.004 a	–0.97 ± 0.12 c	–0.27 ± 0.007 a	–0.70 ± 0.005 b	–0.32 ± 0.05 a	–1.08 ± 0.09 c	0.02	<0.001	0.096
Leaf [ABA] (ng g^–1^ DW)	243 ± 9 b	547 ± 30 a	252 ± 5 b	602 ± 59 a	228 ± 59 a	660 ± 55 a	0.301	<0.001	0.234
Leaf [OPDA] (ng g^–1^ DW)	27 ± 4 cd	34 ± 5 c	54 ± 2 b	83 ± 7 a	14 ± 2 de	11 ± 5 e	0.007	<0.001	0.009
Leaf [JA] (ng g^–1^ DW)	13 ± 1 bc	49 ± 3 a	15.5 ± 1.6 b	17 ± 2 b	5.1 ± 0.5 d	8.1 ± 0.9 cd	<0.001	<0.001	<0.001
Leaf [JA-Ile] (ng g^–1^ DW)	2.7 ± 0.2 b	15 ± 1 a	2.1 ± 0.1 bc	3.5 ± 0.6 b	0.4 ± 0.1 c	0.62 ± 0.01 c	<0.001	<0.001	<0.001
Xylem sap [ABA] (nM)	189 ± 15 c	2775 ± 386 a	193 ± 15 c	2178 ± 284 ab	197 ± 32 c	1894 ± 409 b	0.253	<0.001	0.242
Xylem sap [OPDA] (nM)	58 ± 7 cd	85 ± 7 bc	96 ± 78 b	164 ± 14 a	30 ± 13 d	54 ± 7 d	<0.001	<0.001	0.081
Xylem sap [JA] (nM)	95 ± 8 cd	385 ± 20 a	119 ± 17 b	152 ± 21 b	31 ± 5 de	58 ± 11 de	<0.001	<0.001	<0.001
Xylem sap [JA-Ile] (nM)	1.9 ± 0.2 bc	3.7 ± 0.7 a	1.4 ± 0.2 c	2.5 ± 0.2 b	0.11 ± 0.04 d	1.0 ± 0.2 cd	<0.001	<0.001	0.328
Root [ABA] (ng g^–1^ DW)	247 ± 17 c	1825 ± 320 a	238 ± 320 a	2422 ± 227 a	240 ± 18 c	2025 ± 240 a	0.333	<0.001	0.314
Root [OPDA] (ng g^–1^ DW)	26 ± 3 d	57 ± 3 b	46 ± 3 c	68 ± 7 a	9 ± 1 e	16 ± 2 de	<0.001	<0.001	0.016
Root [JA] (ng g^–1^ DW)	86 ± 13 b	209 ± 29 a	62 ± 3 bc	40 ± 4 cd	8 ± 2 d	13 ± 3 de	<0.001	0.005	<0.001
Root [JA-Ile] (ng g^–1^ DW)	13 ± 2 b	23 ± 3 a	8 ± 1 bc	11 ± 1 b	1.3 ± 0.6 d	3.6 ± 0.6 cd	<0.001	0.002	0.074

Values are the mean ±SE of four replicates, while different letters denote significant (*P*<0.05) differences across genotypes and treatments. The table summarizes significance (*P*-values) of genotype (G), treatment (T), and their interaction (G×T) after ANOVA

## Results

### Ungrafted plants

Under well-watered (WW) conditions, transpiration rates of WT and *spr2* plants were statistically similar, while the *def-1* transpiration rate was 35% lower (Table 1). Under water deficit (WD) conditions, all three genotypes had a similar transpiration rate. Stomatal density and size were inversely correlated, with stomatal density of WT plants 32% and 42% higher than that of *def-1* and *spr2* plants, respectively, while stomatal size of WT plants was 36% and 52% lower than that of *def-1* and *spr2* plants (see Supplementary Fig. S1 at *JXB* online). Under WW conditions, all genotypes had a similar leaf water potential (Ψ_leaf_), but, under WD conditions, Ψ_leaf_ of *def-1* plants was higher than in the WT and *spr2* (–0.7, –0.97, and –1.08 MPa, respectively). These differences in shoot water relations were not due to differences in SWC (data not shown).

WT, *def-1*, and *spr2* plants had similar tissue ABA concentrations under both WW and WD conditions. Water deficit increased root and leaf ABA concentrations by 8.7- and 2.5-fold, respectively (averaged across all genotypes). Although leaf xylem sap ABA concentrations under WW conditions did not differ between genotypes, under WD conditions xylem ABA concentrations of *def-1* and *spr2* plants were 22% and 32% lower than that of WT plants, respectively. This genetic variation in ABA status did not affect transpiration.

OPDA concentrations differed significantly between treatments and genotypes. Under WW conditions, root OPDA concentrations of *def-1* and *spr2* were 70% higher and 70% lower than those of the WT, respectively. Water deficit stimulated root OPDA accumulation by 1.8-fold (averaged across all genotypes). Likewise, water deficit stimulated foliar OPDA accumulation in WT and *def-1* plants, but not in *spr2* plants. Xylem sap OPDA concentrations showed similar responses, which were 78% higher in *def-1* but 42% lower in *spr2* (averaged across water treatments). Water deficit increased xylem OPDA concentrations by 65% (averaged across genotypes).

Under WW conditions, the WT and *def-1* had similar tissue and xylem sap JA concentrations, whereas *spr2* had significantly lower (by 60–80%) JA concentrations than the WT. In WT plants, water deficit increased root, leaf xylem sap, and leaf JA concentrations by 2.4-, 4-, and 3.8-fold, respectively. However, water deficit did not significantly increase JA accumulation in *def-1* and *spr2* plants. JA-Ile concentrations showed similar genotypic and treatment differences to JA, except that xylem JA-Ile concentration significantly increased in all genotypes, but with lower absolute values in both mutants.

Since *def-1* plants had similar leaf area but different transpiration responses to WT plants (Table 1), the role of differential oxylipin accumulation and root and shoot regulation of plant water relations was investigated by reciprocally grafting WT and *def-1* plants. Moreover, these graft combinations allowed the physiological effects of attenuated water deficit-induced JA accumulation to be assessed independently of constitutively low oxylipin status.

### Grafted plants

Under WW conditions, hormonal imbalance of *def-1* self-grafts decreased whole-plant transpiration by 43% compared with WT self-grafts (Fig. 1). Both reciprocal graft combinations had intermediate, similar whole-plant transpiration rates, which were 25% lower than that of WT self-grafts. Thus hormonal imbalance in either scion or rootstock significantly (*P*<0.001 for rootstock and scion main effects) decreased whole-plant transpiration.

Stomatal conductance (*g*_s_) declined linearly with SWC in all graft combinations (Fig. 2A, B). Sensitivity to soil drying (defined as the slope of *g*_s_*versus* SWC) was generally similar in all graft combinations (except *def-1* self-grafts) once *g*_s_ values were normalized according to the maximum *g*_s_ of each graft combination (Fig. 2C). Although *g*_s_ of *def-1* self-grafts was less sensitive to drying soil, all graft combinations had similar absolute *g*_s_ values when the soil profile was depleted of soil moisture. Thus the *def-1* genotype as either scion or rootstock significantly decreased maximum *g*_s_, but only *def-1* self-grafts had diminished stomatal sensitivity to drying soil.

Under WW conditions, all graft combinations had similar values of leaf water potential [Ψ_leaf_ ranged between –0.4 MPa and –0.55 MPa (Fig. 3A)] and root water potential [Ψ_root_ ranged between –0.01 (with some plants showing spontaneous exudation with no applied pressure) and –0.19 MPa]. Root water potential declined exponentially as the soil dried, but similarly in all graft combinations (Fig. 3B). Leaf water potential declined linearly with SWC (Fig. 3A), but more so in WT scions (significant scion×SWC interaction). Taken together, this implies that drought-induced stomatal closure in *def-1* scions (Fig. 2B) was more effective at regulating Ψ_leaf_ than in WT scions (Fig. 3A).

Stomatal conductance decreased linearly with Ψ_leaf_ in WT scions (Fig. 4A), but exponentially in *def-1* scions (Fig. 4B). The rootstock had no impact on the sensitivity of *g*_s_ to Ψ_leaf_ in either *def-1* or WT scions (once *g*_s_ values were normalized according to the maximum *g*_s_ of each graft combination).

Root hydraulic conductance (L_pr_), calculated as the slope of sap flow from de-topped root systems versus applied pressures and normalized to root surface area, was entirely rootstock dependent (Fig 5A). L_pr_ of WT rootstocks was approximately double that of *def-1* rootstocks (Fig. 5B).

Foliar ABA concentration increased with soil drying in all graft combinations (Fig. 6A), but WT scions accumulated significantly more ABA in leaves of plants at low SWC (significant scion×SWC interaction). When exposed to drying soil, ABA accumulation in WT scions was 24% and 50% higher than in the *def-1*/WT (scion/rootstock) and *def-1/def-1* graft combinations, respectively. Although a *def-1* rootstock had no significant impact on drought-induced ABA accumulation in WT scions, a WT rootstock enhanced ABA accumulation in *def-1* scions (significant scion×rootstock interaction). Stomatal closure was correlated with foliar ABA accumulation (Fig. 7A), but was more sensitive in *def-1* scions, which accumulated half as much ABA at maximal stomatal closure.

Foliar JA concentration also increased with soil drying in all graft combinations except *def*-1 self-grafts (Fig. 6B). JA accumulation in WT scions was double that of the *def-1*/WT graft combination, while *def-1* self-grafts did not accumulate JA when exposed to drying soil. Rootstock genotype did not significantly affect JA accumulation in WT scions, but a WT rootstock significantly increased drought-induced JA accumulation in *def-1* scions (significant rootstock×SWC interaction). JA concentration increased with stomatal closure in WT scions (Fig. 7B), whereas this trend neared significance (*P*=0.065) in the *def-1*/WT graft combination. Although absolute JA/JA-Ile concentrations were ~5-fold lower in all graft combinations compared with the experiments with ungrafted plants (cf. Table 1), these were probably due to differences in analytical (instrument) sensitivity, and both experiments showed similar treatment and genotypic relative differences.

Soil drying induced a similar pattern of JA-Ile accumulation in the graft combinations as JA (Fig. 6D). JA-Ile accumulation in WT scions was 38% higher than that of the *def-1*/WT graft combination, while *def-1* self-grafts did not accumulate JA-Ile when exposed to drying soil. However, rootstock genotype did not affect JA-Ile accumulation in WT or *def-1* scions. Only in WT scions did JA-Ile concentrations increase with stomatal closure (Fig. 7D).

Whereas soil drying induced only limited (36% increase) foliar OPDA accumulation in WT self-grafts and had no effect in WT/*def*-1 plants (Fig. 6C), soil drying substantially increased OPDA concentrations of *def-1* scions (significant scion×SWC interaction). WT rootstocks decreased OPDA accumulation in *def-1* scions (significant scion×rootstock×SWC interaction), such that foliar OPDA concentrations were 44% lower than in *def-1* self-grafts. Stomatal conductance was not correlated with OPDA concentration in WT scions, but leaf OPDA concentrations increased with stomatal closure in *def-1* scions (Fig. 7C).

Water deficit decreased sap flow of all graft combinations (Table 2); however, plants with a *def-1* scion had significantly lower flow rates than plants with a WT scion (the significance, *P*-values, of yhr flow rate and hormone delivery are summarized in Table 3). All graft combinations had similar root xylem ABA concentrations under both WW and WD conditions, with water deficit increasing xylem ABA concentrations by 11.5-fold from 37 nM to 426 nM (averaged across graft combinations). Xylem JA concentrations were similar in all graft combinations under WW conditions (48 nM); however, water deficit significantly increased JA concentrations of graft combinations with a WT rootstock (to 1030 nM). JA-Ile followed a similar trend, with low concentrations under WW conditions but higher JA-Ile concentrations when graft combinations with a WT rootstock dried the soil.

**Table 2.** Xylem sap flow rate, root xylem sap hormone concentrations (ABA/JA/JA-Ile), and hormone delivery (the product of sap flow rate and hormone concentration) under well-watered (WW) and water deficit (WD) conditions in the wild type (WT/WT) and *def-1* (*def-1/def-1*) self-grafts and reciprocal grafts (WT/*def-1*, *def-1*/WT)

	WT/WT		WT/*def*-1			*def-1*/WT	*de*f*-1*/*def-1*	
	WW	WD	WW	WD	WW	WD	WW	WD
Flow (nmol s^–1^)×100	4.8 ± 0.4 a	1.8 ± 0.3 d	3.4 ± 0.9 b	1.6 ± 0.3 de	3.0 ± 0.5 b	1.4 ± 0.2 ef	2.6 ± 0.4 c	1.1 ± 0.1 f
[ABA] (nM)	44 ± 5 b	427 ± 35 a	34 ± 4b	378 ± 44 a	31 ± 2 b	475 ± 52 a	38 ± 2 b	538 ± 48 a
[JA] (nM)	52 ± 11 bc	1047 ± 154 a	57 ± 9 bc	598 ± 68 b	43 ± 7 bc	1014 ± 69 a	42 ± 5 c	426 ± 42 bc
[JA-Ile] (nM)	4.1 ± 0.2 c	73 ± 9 a	2.7 ± 0.9 c	45 ± 13 ab	3.9 ± 0.6 c	66 ± 15 a	6.4 ± 1.4 c	26 ± 11 b
ABA delivery (nmol s^–1^)×10^5^	3.8 ± 0.2 b	14.2 ± 0.1 a	2.1 ± 0.2 b	11 ± 1 a	1.7 ± 0.2 b	11.9 ± 0.2 a	1.4 ± 0.1 b	10.7 ± 0.9 a
JA delivery (nmol s^–1^)×10^5^	4.5 ± 0.6 d	35 ± 5 a	3.5 ± 0.9 d	18 ± 3 b	5.4 ± 0.7 d	25 ± 4 b	4.0 ± 0.4 d	8.5 ± 2.3 c
JA delivery (nmol s^–1^)×10^5^	0.3 ± 0.1 b	2.4 ± 0.4 a	0.2 ± 0.1 b	1.3 ± 0.5 a	0.2 ± 0.1 b	1.6 ± 0.4 a	0.2 ± 0.1 b	0.5 ± 0.2 b

Values are the mean ± SE of four replicates. Letters denote significant (*P*<0.05) differences across genotype and treatment after a post-hoc (Duncan’s HSD).

**Table 3.** A summary of the significance (*P*-values) of rootstock genotype (R), scion genotype (S), treatment (T), and interactions on sap flow and hormone delivery after ANOVA

	Flow	ABA delivery	JA delivery	JA-Ile delivery
Treatment	<0.001	<0.001	<0.001	<0.001
Root	<0.001	0.042	<0.001	<0.001
Shoot	<0.001	0.062	0.004	0.001
T×R	0.05	0.68	<0.001	<0.001
T×S	0.06	0.2	0.026	0.01
R×S	0.326	0.45	0.44	0.63
T×R×S	0.804	0.26	0.51	0.55

Hormone delivery from the root system was quantified by multiplying hormone concentration and pressure-induced xylem flow (Table 2). Although water deficit increased ABA delivery by an order of magnitude, all graft combinations had similar ABA delivery. In contrast, both treatment and root/shoot genotype affected JA delivery. Although all graft combinations had similar root JA export under WW conditions, water deficit increased root JA export of WT self-grafts, both reciprocal grafts, and *def-1* self-grafts by 7-, 5-, and 2.6-fold, respectively. Root JA-Ile export followed a similar pattern. Thus water deficit always increased root JA/JA-Ile export, even if it had no measurable effect on foliar JA/JA-Ile concentrations (as in *def-1* self-grafts).

### Detached leaf transpiration assays

To determine whether xylem-supplied phytohormones could regulate transpiration, ABA or JA were supplied via the transpiration stream (to emulate root to shoot hormone delivery) to detached WT, *def-1*, and *spr2* leaves. Detached leaf transpiration was more sensitive to xylem-supplied ABA than xylem-supplied JA, with significant reductions at 100 nM and 1000 nM, respectively (Supplementary Fig. S2). As in whole plant measurements, the transpiration rate of detached *def-1* leaves was (21%) lower than that of WT leaves when supplied with an artificial xylem solution, while *spr2* transpiration was significantly (9.6%) higher (Fig. 8). Xylem-supplied JA (1000 nM) decreased transpiration of WT and *spr2* leaves by 16% and 24%, respectively, but increased transpiration of *def-1* leaves by 23%. Xylem-supplied ABA (1000 nM) decreased transpiration in all genotypes, but more effectively in WT and *spr2* (50% reduction) than in *def-1* (26% reduction).

To determine whether xylem-supplied ABA or JA influenced synthesis or metabolism of each other, foliar (Fig. 9) and xylem sap (Supplementary Fig. S3) phytohormone concentrations were quantified at the end of the transpiration assay, after supplying artificial xylem sap via the transpiration stream for 5 h. All three genotypes had similar ABA and JA/JA-Ile concentrations to freshly harvested leaves (pre- and post-excision controls). Leaf OPDA concentrations of WT and *spr2* plants were similar in detached leaves supplied with artificial xylem sap and in freshly harvested leaves. After 5 h of feeding *def-1* leaves with artificial xylem sap, their OPDA concentrations were 4.7-fold higher than those of the other genotypes fed artificial xylem sap, and 2.4-fold higher than freshly harvested *def-1* leaves. Taken together, these data imply a limited effect of leaf excision and placing the cut petiole in an artificial xylem sap for 5 h, as foliar phytohormone concentrations were generally similar to those of freshly harvested leaves that were immediately placed in liquid nitrogen post-excision.

Xylem-supplied ABA more than doubled leaf ABA concentrations of WT and *def-1* plants, but had a much smaller effect in *spr2* leaves (Fig. 9A). Xylem-supplied ABA had no effect on JA and OPDA levels in WT and *spr2* plants, but decreased OPDA concentrations (by 33%) while increasing JA concentrations (2.4-fold) in *def-1* plants (Fig. 9C, E). Xylem-supplied ABA increased JA-Ile concentrations by 5.3-fold, averaged across both genotypes (Fig. 9G). Taken together, xylem-supplied ABA significantly increased JA-Ile concentrations in WT and mutant genotypes, while significantly decreasing OPDA concentrations in *def-1*.

Xylem-supplied JA had no effect on leaf ABA concentration in all genotypes (Fig. 9A), but substantially increased JA and JA-Ile concentrations (Fig. 9E, G). These increases were much greater in WT plants (5.5-fold averaging both JA and JA-Ile) than in *def-1* (3.4-fold) and *spr2* (4-fold). Xylem-supplied JA had no effect on leaf OPDA concentrations in WT and *spr2* plants (Fig. 9C), but decreased OPDA concentrations of detached *def-1* leaves by 51% compared with plants supplied with artificial xylem sap (Fig. 9C, D). Genotypic variation in oxylipin status in leaves supplied with JA via the transpiration stream was not explained by genotypic differences in JA uptake.

Taken together, supplying ABA or JA via the transpiration stream to detached leaves had the intended effects on foliar concentrations of the hormone supplied, but ABA also increased JA-Ile concentrations in all genotypes, and decreased OPDA concentrations in *def-1* plants.

## Discussion

Although exogenous JA induces stomatal closure (Herde *et al.*, 1997; Hossain *et al.*, 2011; Yin *et al.*, 2016), and water deficit stimulates root (De Ollas *et al.*, 2013; 2015) and shoot (Muñoz-Espinoza *et al.*, 2015) JA and JA-Ile (the bioactive form) accumulation, this study uniquely addresses the physiological significance of JA as a long-distance signal of water deficit. Two complementary approaches were used: allowing reciprocally grafted WT and *def-1* tomato plants (which fail to accumulate JA and JA-Ile during water deficit) to dry the soil and supplying detached leaves with xylem-borne JA at the concentrations detected in WT plants grown in drying soil. Although xylem JA concentration of WT plants exposed to water deficit was sufficient to elicit partial stomatal closure when supplied via the xylem to detached WT leaves, attenuating drought-induced root JA export (in WT/*def-1* and *def-1*/WT plants) did not alter stomatal sensitivity to drying soil (Fig. 2C). Only *def-1* self-grafts, with the lowest stomatal conductance under well-watered conditions, showed an attenuated stomatal response to drying soil, consistent with their lowest root JA export. Paradoxically, increasing the supply of xylem-borne JA to *def-1* shoots (in either detached leaves or *def-1*/WT plants) increased transpiration, probably by limiting OPDA (a JA precursor) accumulation, which is a more potent antitranspirant than JA (Savchenko *et al.*, 2014). Although spatio-temporal patterns of JA accumulation differed between detached leaf transpiration assays and grafted plants exposed to drying soil, variation in both foliar and xylem sap oxylipin status mediated both constitutive stomatal conductance and stomatal sensitivity to drying soil.

### Stomatal responses under well-watered conditions

Well-watered, ungrafted *def-1* plants had constitutively lower transpiration rates than either *spr2* or WT plants (Table 1). Since all genotypes had similar leaf water potential and xylem and leaf ABA concentration, stomatal morphology and oxylipin status were measured. Decreased stomatal density of the mutants was compensated by increased stomatal size (Supplementary Fig. S1), making it difficult to attribute variation in transpiration rate to altered stomatal morphology. Alternatively, while *spr2* had lower OPDA, JA, and JA-Ile concentrations than WT plants, foliar OPDA concentrations were almost doubled in *def-1* plants, supporting the suggestion that OPDA is a more potent antitranspirant than JA/JA-Ile (Savchenko *et al.*, 2014).

Although both mutants failed to accumulate JA as the soil dried (Table 1), *def-1* was preferred in reciprocal grafting studies since it had similar JA/JA-Ile concentrations to WT plants under WW conditions. Moreover, *def-1* plants had similar vegetative vigour to WT plants, unlike *spr2* (Table 1; Supplementary Fig. S4), eliminating the need for a developmental control in soil drying experiments. Although the grafting process itself probably induced transient differences in endogenous JA concentrations, which could inhibit leaf expansion (Moore *et al.*, 2003) of WT scions, it was possible to select reciprocally grafted *def-1* and WT plants of similar leaf area to measure transpiration.

Nevertheless, hormonal imbalance in either rootstock or scion decreased transpiration of well-watered plants (Fig. 1) such that grafting WT scions onto *def-1* rootstocks decreased their *g*_s_ compared with WT self-grafts (Fig. 2), while grafting *def-1* scions onto WT rootstocks increased their *g*_s_ compared with *def-1* self-grafts. These rootstock-mediated changes in *g*_s_ were correlated with decreased root hydraulic conductivity of *def-1* rootstocks (Fig. 5) as previously reported (Sánchez-Romera *et al.*, 2014), yet well-watered plants of all graft combinations maintained a similar leaf water potential (Ψ_leaf_) (Fig. 3A). While homeostatic regulation of Ψ_leaf_ by *g*_s_ may co-ordinate root and shoot conductance (Jones, 1998; Ripullone *et al.*, 2007), it seems unlikely that variation in plant hydraulics caused genotypic differences in transpiration of well-watered plants.

### Stomatal responses to drying soil were independent of leaf water status

**Fig. 10.**
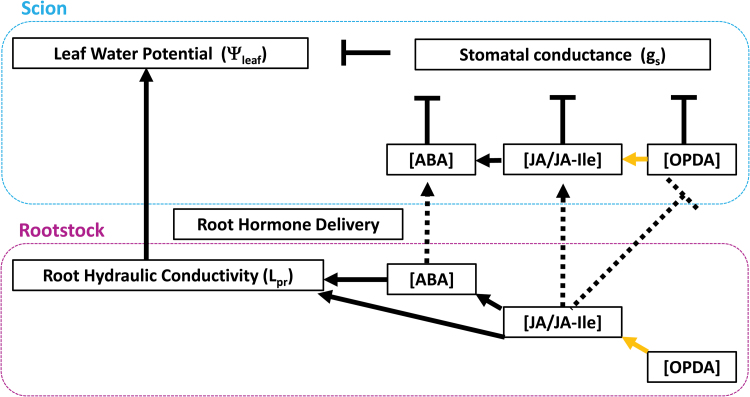
Model of plant hydraulics and phytohormone impacts on stomatal conductance of grafted plants. Lines ending in arrowheads indicate a positive impact, while lines ending in a bar indicate negative impacts. Root hormone delivery is indicated by dashed lines, while relationships within a biosynthetic pathway are indicated by a yellow arrow.

Genetic variation in stomatal sensitivity to drying soil was assessed by normalizing *g*_s_ to the maximum (well-watered) value achieved by each graft combination (Fig. 2C). Only *def-1* self-grafts (which failed to accumulate JA and its bioactive conjugate JA-Ile as the soil dried) were relatively insensitive to soil drying. Thus WT levels of JA/JA-Ile are required (in either rootstock or scion) to maximize stomatal sensitivity to drying soil, suggesting an antitranspirant effect of JA (Herde *et al.*, 1997). Nevertheless, perturbed leaf hydraulics has been suggested as the most likely mechanism causing stomatal closure (Tardieu, 2016). Although soil drying decreased Ψ_leaf_ in all graft combinations, *def-1* scions maintained a higher Ψ_leaf_ than WT scions (Fig. 3A), independent of root hydraulic conductivity (lower in *def-1* rootstocks, irrespective of scion; Fig. 5). This implies that stomatal conductance regulates Ψ_leaf_ (rather than vice versa), as in previous tomato experiments where higher *g*_s_ was associated with decreased Ψ_leaf_ in WT plants exposed to various irrigation treatments (Kudoyarova *et al.*, 2007) and in reciprocally grafted, well-watered WT and ABA-deficient plants (Dodd *et al.*, 2009). Taken together, these data imply that phytohormones regulate stomatal closure in response to soil drying (Fig. 10).

### Phytohormonal (ABA and JA/JA-Ile) regulation of stomatal response to drying soil

Although an increased foliar and xylem sap ABA concentration has been correlated with stomatal closure in multiple species including tomato (Dodd, 2007; Thompson *et al.*, 2007), *def-1* scions required half the foliar ABA concentration to induce maximal stomatal closure (Fig. 7A). Possibly *def-1* scions were more sensitive to ABA, but similar stomatal closure of *def-1* and WT leaves in response to xylem-borne ABA (Fig. 8; Supplementary Fig. S2) disproves this hypothesis. Stomatal conductance responds more sensitively to xylem sap ABA concentration than foliar ABA accumulation (Geilfus *et al.*, 2015; Tardieu *et al.*, 2015), as much of the latter is compartmentalized in mesophyll cells and is unavailable to the guard cells (Wilkinson *et al.*, 1998). While grafting *def-1* scions onto WT rootstocks increased foliar ABA accumulation compared with *def-1* self-grafts (Fig. 6A), xylem ABA concentration of *def-1* scions was rootstock independent and similar to that of WT scions (Table 2). Taken together, the similar increases in xylem ABA concentration in all graft combinations, yet attenuated stomatal response to drying soil in *def-1* self-grafts (Fig. 2C), suggests that other phytohormones were involved in regulating this response.

Restoring normal (WT) stomatal sensitivity to drying soil by grafting *def-1* scions onto WT rootstocks was correlated with greater root JA export (Table 2) and partial restoration of foliar JA and JA-Ile status (Fig. 6B, D). Nevertheless, supplemental JA feeding to detached *def-1* leaves actually increased their transpiration (Fig. 8), consistent with the higher transpiration of *def-1*/WT plants than *def-1* self-grafts (Fig. 1). Thus stomatal regulation by JA seems dependent on tissue water status, since normal JA levels are required for maximal stomatal conductance in well-watered plants, while drought-induced JA accumulation enhances stomatal closure. Future experiments should establish whether this foliar JA accumulation is a local (induced by decreased Ψ_leaf_) or long-distance (increased root export of JA; Table 2) signal, especially since the xylem JA concentrations detected in WT plants induced partial stomatal closure when fed via the xylem to detached leaves (Fig. 8).

### OPDA affects stomatal responses of plants to drying soil

Endogenous OPDA concentrations are rarely monitored in plants exposed to water deficit, although they increase even in WT self-grafts (Fig. 6C), and by up to an order of magnitude (Seo *et al.*, 2001; Arbona *et al.*, 2010; Grebner *et al.*, 2013). With soil drying, OPDA accumulation was inversely related to JA accumulation across all graft combinations (cf. Fig. 6B, C), with maximal OPDA levels in *def-1* self-grafts. Grafting *def-1* scions onto a WT rootstock increased root JA export (Table 2) and decreased foliar OPDA concentrations (Fig. 6C), consistent with suggestions that JA treatment or transport from distal tissues decreases OPDA concentration (Scalschi *et al.*, 2015) by negatively regulating jasmonate biosynthesis or metabolism (Kazan and Manners, 2008). Thus root to shoot JA transport may directly affect transpiration of WT plants (Fig. 1), but may also indirectly regulate transpiration of *def-1* scions by decreasing foliar OPDA concentrations (Fig. 6C).

To test the hypothesis that xylem-borne JA/JA-Ile mediates transpiration by limiting foliar OPDA accumulation, JA was supplied via the xylem to detached *def-1* and WT leaves. Xylem-supplied JA had opposite effects on both transpiration and foliar OPDA concentrations in the two genotypes. Although xylem-borne JA decreased transpiration of WT leaves, it was significantly less effective than ABA supplied at the same concentrations. In contrast, xylem-supplied JA increased transpiration of *def-1* leaves (Fig. 8), consistent with the partial rescue of transpiration in *def-1* scions grafted to WT roots (Fig. 1). Whereas xylem-borne JA had no effect on OPDA concentrations in WT leaves, in *def-1* leaves increased transpiration was associated with decreased OPDA (high antitranspirant effect) concentrations and increased JA and *in planta* synthetized JA-Ile (low antitranspirant effect) concentrations (Fig. 9). Taken together, this supports a model in which xylem-transported JA significantly influences *g*_s_ by regulating accumulation of the JA precursor OPDA (Fig. 10).

### Conclusions

Although spatio-temporal patterns of JA accumulation differed between detached leaf transpiration assays and grafted plants exposed to drying soil, additional xylem-borne JA consistently decreased stomatal conductance of WT shoots (by activating JA signalling through the COI receptor) while increasing stomatal conductance of *def-1* shoots (by limiting OPDA accumulation which acts via a COI1-independent pathway). Further work is required to establish the relative importance of these two signalling pathways in WT plants and the distal physiological effects of root to shoot jasmonate transport in plants exposed to water deficit.

## Supplementary Material

Supplementary Figures and Tables
