# Supplementary material for: Attenuated accumulation of jasmonates modifies stomatal responses to water deficit
Source: J Exp Bot. 2018 Feb 8;69(8):2103–16. doi: 10.1093/jxb/ery045 (PMC6018964; doi:10.1093/jxb/ery045)
Supplement: Supplementary Figures and Tables [file ery045_suppl_supplementary_figures_and_tables.pdf]

Supplemental data:

S1. Representative microscopy images of epidermal strips of WT (cv. Castlemart – CST) (upper row), *def-1* (middle row) and *spr2* (lower row). Histograms represent stomatal density and size of at least 30 random sections of each genotype. Bars are mean ± SE, with different letters denoting significant differences between genotypes after Duncan´s test.

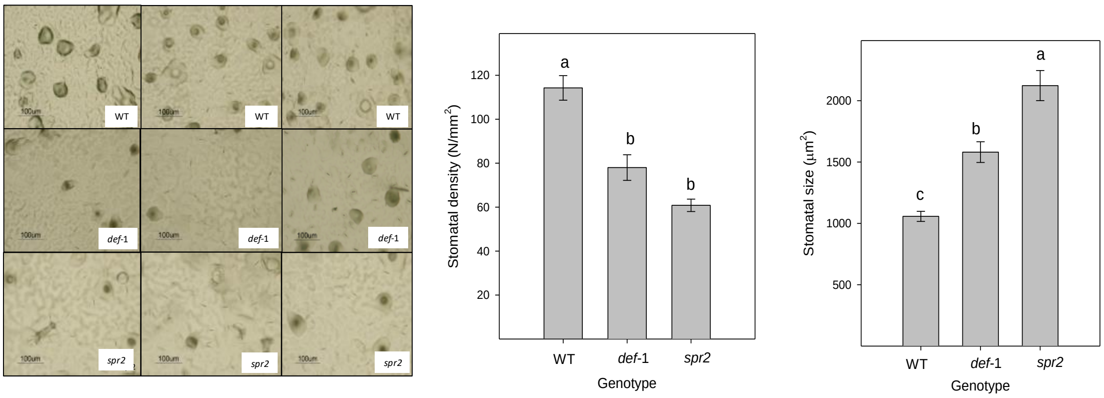

S2. Transpiration rate of detached WT *(CST)* and *def-1* leaves fed with artificial xylem sap with 10, 100 and 1000 nM ABA (black bars) and JA (grey bars). Bars are mean ± SE of 4 replicates, while asterisks denote significant differences between treatments and 0 nM. Table summarizes significance (P Values) of genotype, hormone treatment and concentration with interactions after ANOVA.

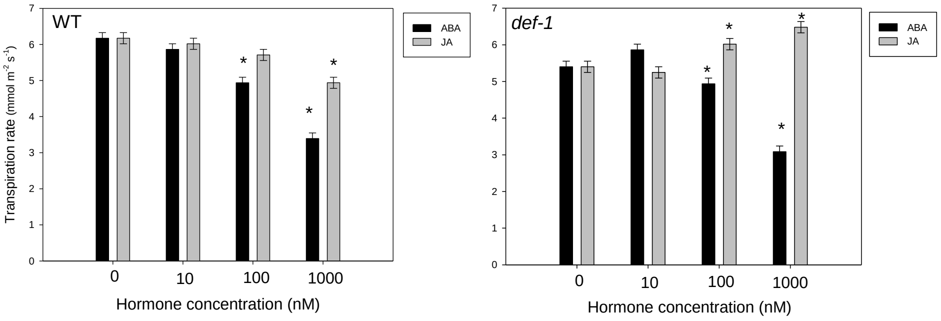

Factor	p Value
Genotype	0.057
Treatment	<0.001
Concentration	<0.001
G x T	0.033
G x C	<0.001
T x C	<0.001
G x T x C	<0.001

S3. ABA **(A, B)** OPDA **(C, D)**, JA **(E, F)** and JA-Ile **(G, H)** concentrations of WT, *def-1* and *spr2* leaf xylem sap after 5 hours of xylem feeding with artificial xylem sap (hollow bars), 1000 nM ABA (grey bars) or 1000 nM JA dark grey bars (left panels) or freshly detached leaves before (filled bars) and 5 hours after (grey bars) the transpiration assay (right panels). Bars are mean ± SE of 4 replicates, with different letters denoting significant differences between treatments after Duncan´s test, comparing across both panels. Tables summarise significance (P Values) of genotype, treatment and their interactions after ANOVA.

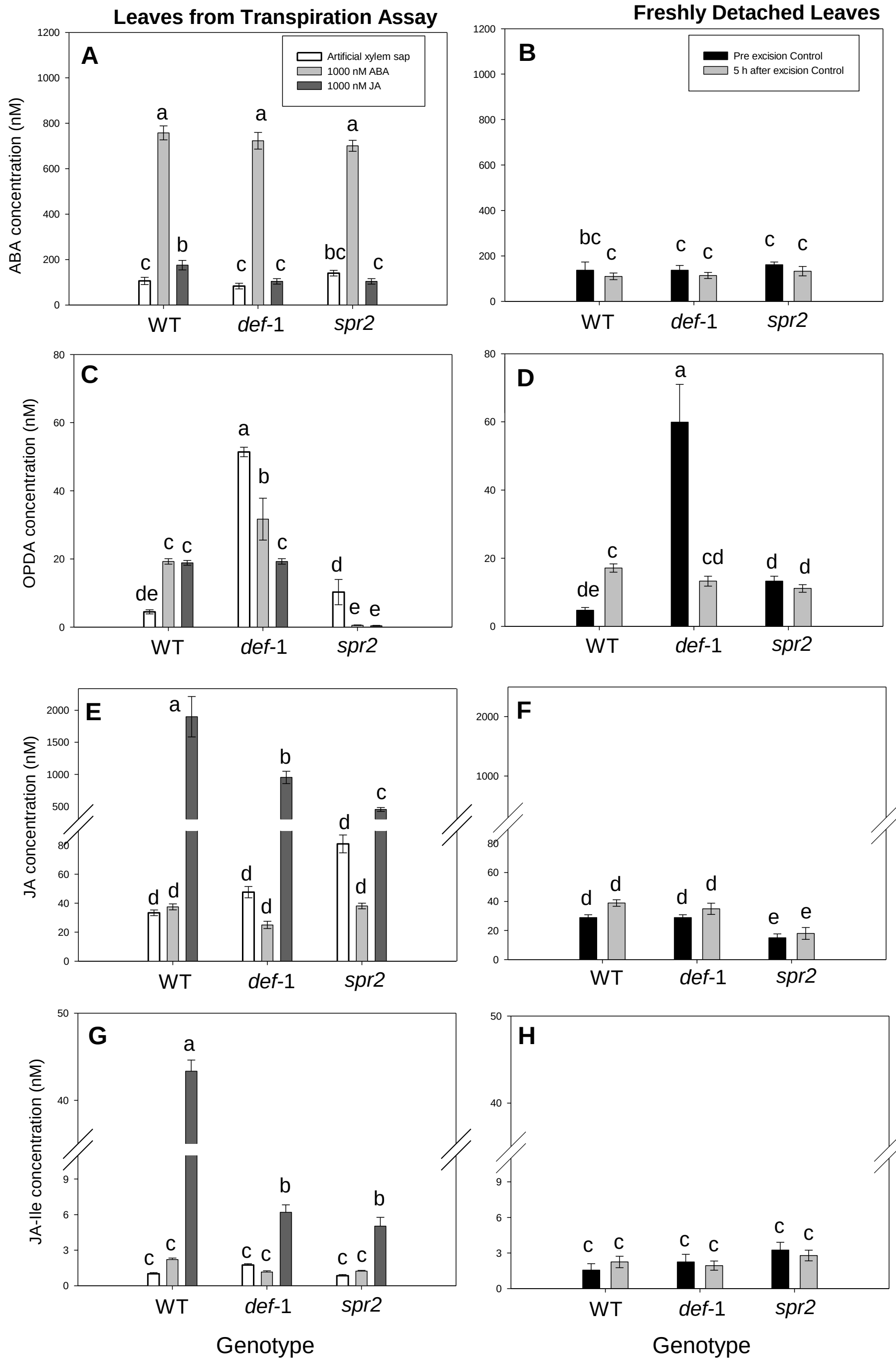

Factor	p Value
Genotype	0.056
Treatment	<0.001
G x T	0.132

Factor	p Value
Genotype	<0.001
Treatment	<0.001
G x T	<0.001

Factor	p Value
Genotype	<0.001
Treatment	<0.001
G x T	<0.001

Factor	p Value
Genotype	<0.001
Treatment	<0.001
G x T	<0.001

S4. Representative images of three-week-old **A)** *spr2*, **B)** Wild-Type (WT) and **C)** *def*-1 genotypes **D)** Comparative of the three-week-old genotypes and **E)** Comparative six-weeks-old genotypes before harvesting.

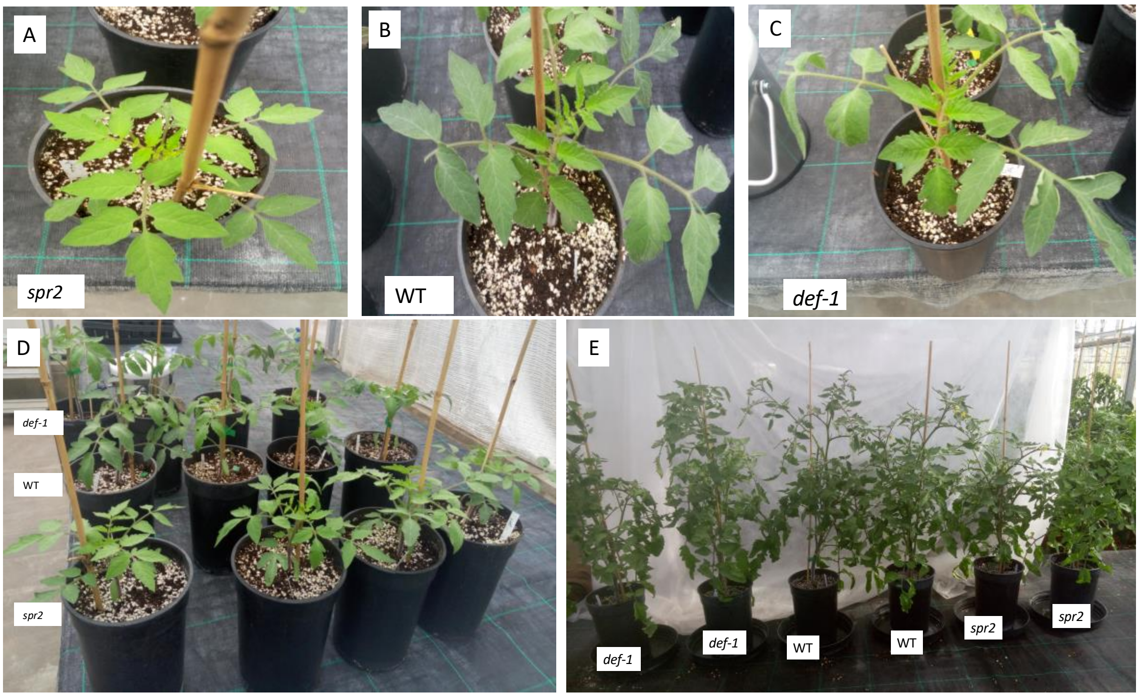
A
spr2
B
WT
C
def-1
D
def-1
WT
spr2
E
def-1
def-1
WT
WT
spr2
spr2
